# Clinical outcomes of severe sepsis and septic shock patients with left ventricular dysfunction undergoing continuous renal replacement therapy

**DOI:** 10.1038/s41598-022-13243-9

**Published:** 2022-06-07

**Authors:** Guangwei Yu, Kun Cheng, Qing Liu, Wenwei Wu, Huashan Hong, Xiaohong Lin

**Affiliations:** 1 Department of Emergency, Fujian Medical University Union Hospital, Fuzhou, Fujian China; 2 Department of Intensive Care Unit, Fujian Provincial Hospital, Fuzhou, Fujian China; 3 Fujian Critical Care Medicine Center, Fuzhou, Fujian China; 4 Department of Geriatrics, Fujian Medical University Union Hospital, Fuzhou, Fujian China; 5 Fujian Provincial Clinical College of Fujian Medical University, Fuzhou, Fujian China; 6 Fujian Key Laboratory of Vascular Aging, Fujian Medical University, 29 Xinquan Rd., Fuzhou, 350001 Fujian China

**Keywords:** Cardiology, Diseases

## Abstract

Baseline left ventricular (LV) dysfunction is associated with subsequent risks of acute kidney injury (AKI) and mortality in patients with sepsis. This study investigated the therapeutic effects of continuous renal replacement therapy (CRRT) in hemodynamically unstable patients with severe sepsis and septic shock combined with LV dysfunction. In this multicenter retrospective study, severe sepsis and septic shock patients with LV dysfunction were classified into one of two groups according to the timing of CRRT: the early group (before AKI was detected) or the control group (patients with AKI). Patients from the control group received an accelerated strategy or a standard strategy of CRRT. The primary outcome was all-cause intensive care unit (ICU) mortality. Patients were weighted by stabilized inverse probability of treatment weights (sIPTW) to overcome differences in baseline characteristics. After sIPTW analysis, the ICU mortality was significantly lower in the early group than the control group (27.7% vs. 63.5%, *p* < 0.001). Weighted multivariable analysis showed that early CRRT initiation was a protective factor for the risk of ICU mortality (OR 0.149; 95% CI 0.051–0.434; *p* < 0.001). The ICU mortality was not different between the accelerated- and standard-strategy group (52.5% vs. 52.9%, *p* = 0.970). Early CRRT in the absence of AKI is suggested for hemodynamically unstable patients with severe sepsis and septic shock combined with LV dysfunction since it benefits survival outcomes.

## Introduction

Sepsis is associated with life-threatening multiorgan dysfunction due to the extreme host response to infection^1^. It has become a major global health problem leading to approximately five million deaths annually^2^. Cardiac dysfunction has been identified as a serious component of sepsis-induced organ dysfunction and is observed in 10–70% of patients with a mortality rate as high as 70%^3,4^. Left ventricular (LV) dysfunction is associated with the subsequent risk of acute kidney injury (AKI) under different clinical circumstances^5,6^. For patients with sepsis, LV diastolic dysfunction (LVDD) and LV systolic dysfunction (LVSD) have been reported to worsen renal outcomes^6^. Our previous study revealed that LVDD was associated with septic AKI, and E/e′ and e′ were useful predictors of septic AKI among patients with severe sepsis or septic shock^7^.

Continuous renal replacement therapy (CRRT) is the predominant form of renal replacement therapy (RRT) applied in the intensive care unit (ICU) for the clearance of cytokines and endotoxins, the correction of acid–base and electrolyte disturbance, and to achieve hemodynamic stability^8,9^. The treatment goals for acute heart failure (AHF) in the ICU are to improve organ perfusion and hemodynamic stability, alleviate symptoms, and limit cardiac and renal injury^10^, which can be achieved by CRRT. The CRRT mimics urine output by continuously and slowly removing the plasma water and achieving accurate volume control and hemodynamic stability^9,11^. The 2016 European Society of Cardiology guidelines recommended the consideration of RRT in patients with AHF with refractory volume overload and AKI^10^. CRRT and diuretics showed an equivalent and beneficial effect in relieving clinical signs and symptoms of heart failure but only CRRT was able to improve several instrumental and humoral indicators of congestion^12^. For patients with sepsis or septic shock complicated with AKI who need RRT, there a weak recommendation for continuous or intermittent RRT^1^. For patients with septic shock, CRRT was suggested to facilitate management of fluid balance according to the International Guidelines for Management of Sepsis and Septic Shock: 2012/2016^13,14^. Cardiac dysfunction exacerbates the hemodynamic instability and contributes to renal hypoperfusion^7^. Whether to initiate CRRT in patients with severe sepsis and septic shock complicated with hemodynamic instability before the onset of AKI has not been discovered.

Hence, the clinical outcomes of CRRT before and after AKI in hemodynamically unstable sepsis patients combined with LV dysfunction were investigated in the current study.

## Results

### Patient characteristics before sIPTW

**Figure 1:**
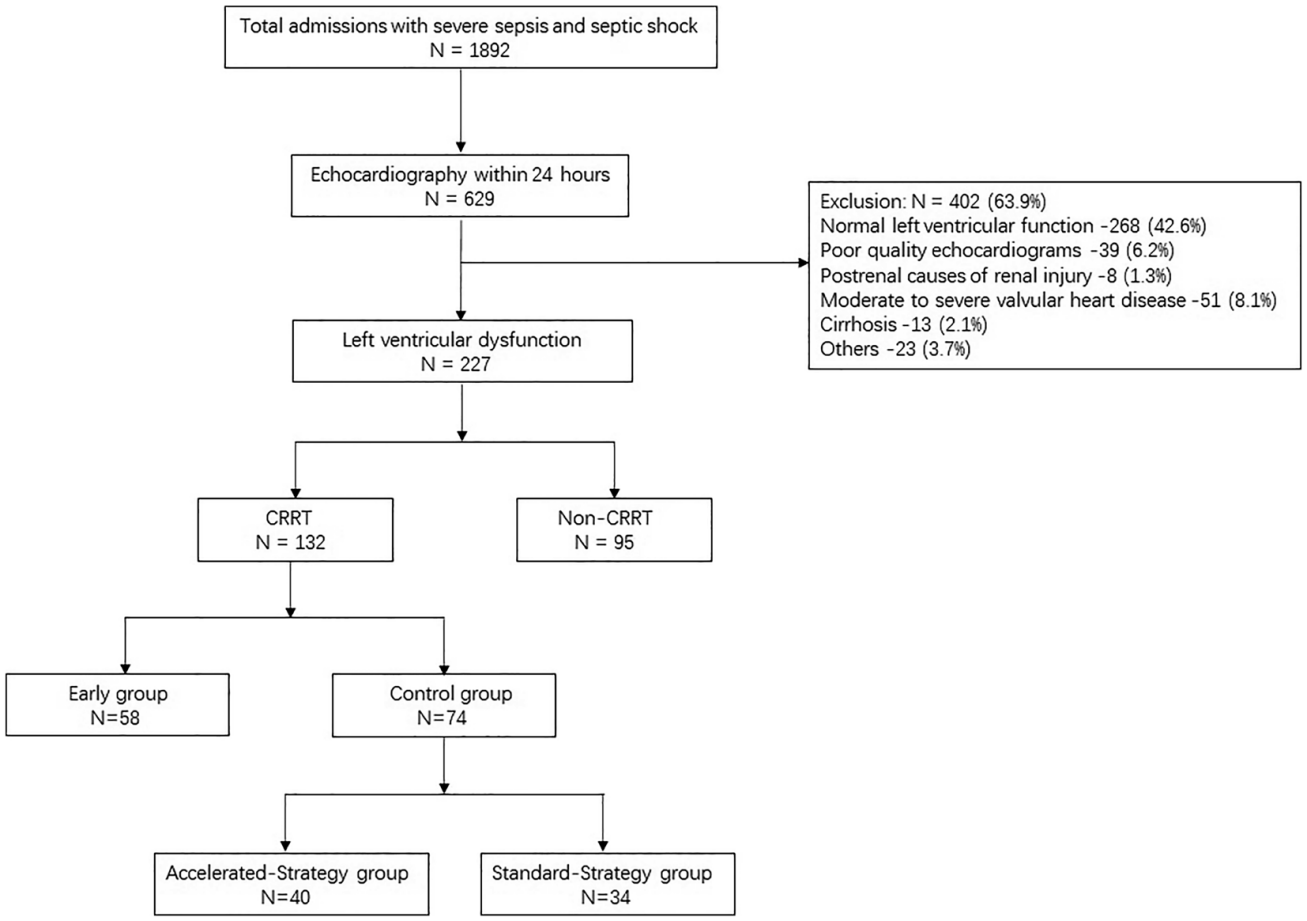
Patient and analysis flowchart. *CRRT* continuous renal replacement treatment.

The patient characteristics, echocardiographic parameters, and CRRT protocol between three centers were presented in Table S1 (additional file). A total of 1892 adult patients with severe sepsis and septic shock were initially screened and 629 patients had echocardiograms performed. Among 227 patients who met the inclusion criteria with LV dysfunction, 132 patients received CRRT. Thirty-seven, 71 and 24 patients had LVSD, LVDD, and combined LVSD and LVDD, respectively. A total of 58 patients received early initiation of CRRT due to unstable hemodynamics and 74 patients were categorized into the control group. Forty patients received an accelerated strategy for the initiation of CRRT and 34 patients received a standard strategy in the control group. The study flowchart was displayed in Fig. 1.

The patient characteristics and echocardiographic parameters before sIPTW were presented in Table 1. The early group had a greater proportion of postoperative patients (25.86% vs. 5.41%, *p* = 0.001) and abdominal surgery (15.52% vs. 4.05%, *p* = 0.023). The control group had worse LV diastolic function as demonstrated by the higher E/e′ (13.26 ± 5.86 vs. 9.98 ± 3.88, *p* < 0.001). Patients with early CRRT had a higher proportion of norepinephrine users at admission (82.76% vs. 66.22%, *p* = 0.033), lower CRRT dose prescription (26.52 ± 2.1 mL/kg/h vs. 29.43 ± 3.46 mL/kg/h, *p* < 0.001) and lower dialysis dose prescription (15.50 ± 1.78 mL/kg/h vs. 20.00 ± 3.58 mL/kg/h, *p* < 0.001).

**Table 1:** Patient characteristics between early group and control group before sIPTW (at baseline and at the start of CRRT).

Characteristics	Early group (n = 58)	Control group (n = 74)	*p*
Age, years	62.86 ± 10.58	62.58 ± 8.87	0.868
Male, n (%)	40 (68.97)	50 (67.57)	0.864
BMI, kg/m^2^	24.34 ± 3.40	24.76 ± 3.19	0.466
MAP, mmHg	81.74 ± 5.38	83.24 ± 5.33	0.112
**Laboratory tests**			
Leukocyte count, × 10^9^/L	14.23 ± 3.71	15.36 ± 3.37	0.070
Neutrophil percentage, %	81.13 ± 7.78	80.93 ± 7.20	0.879
PLT count, × 10^9^/L	178.93 ± 57.44	177.72 ± 54.13	0.901
Blood pH	7.33 ± 0.06	7.34 ± 0.06	0.246
Baseline creatinine, mg/dL	0.93 ± 0.29	0.94 ± 0.30	0.842
Serum potassium, mEq/L	4.76 ± 0.66	4.84 ± 0.68	0.472
Baseline eGFR, mL/min/1.73 m^2^	80.88 ± 22.81	80.05 ± 22.05	0.832
BUN, mg/dL	15.96 ± 2.88	15.91 ± 3.13	0.918
CK-MB, IU/L	27.50 ± 22.75	21.12 ± 14.81	0.067
ALT, U/L	56.24 ± 58.65	53.04 ± 51.45	0.739
Total bilirubin, mg/dL	1.30 ± 0.88	1.20 ± 0.98	0.559
Lactate, mg/dL	72.46 ± 48.73	58.70 ± 32.57	0.067
Hb, g/L	132.64 ± 12.05	131.19 ± 12.76	0.508
Six-hour UO at admission, mL	421.36 ± 151.25	460.50 ± 139.35	0.125
**Primary diagnosis**			
Pneumo-sepsis, n (%)	19 (32.76)	23 (31.08)	0.837
Urosepsis, n (%)	7 (12.07)	5 (6.76)	0.292
Abdominal sepsis, n (%)	18 (31.03)	28 (37.84)	0.416
Other cause, n (%)	14 (24.14)	18 (24.32)	0.980
**Type of surgery**			
Abdominal surgery, n (%)	9 (15.52)	3 (4.05)	0.023
Urinary surgery, n (%)	2 (3.45)	0 (0)	0.191
Others, n (%)	4 (6.9)	1 (1.35)	0.168
Postoperative, n (%)	15 (25.86)	4 (5.41)	0.001
Positive blood culture, n (%)	25 (43.10)	30 (40.54)	0.767
Invasive MV, n (%)	32 (55.17)	47 (63.51)	0.332
**Comorbidities**			
Hypertension, n (%)	14 (24.14)	19 (25.68)	0.840
Diabetes mellitus, n (%)	9 (15.52)	12 (16.22)	0.913
Coronary artery disease, n (%)	3 (5.17)	5 (6.76)	> 0.999
Heart failure, n (%)	7 (12.07)	9 (12.16)	0.987
**Medication at admission**			
Noradrenaline, n (%)	48 (82.76)	49 (66.22)	0.033
Dopamine, n (%)	17 (29.31)	12 (16.22)	0.071
Glucocorticoid, n (%)	6 (10.34)	13 (17.57)	0.241
Digitalis during hospitalization, n (%)	10 (17.24)	11 (14.86)	0.711
Resuscitation fluid in first 36 h (crystal solution), mL	3083.62 ± 457.34	3064.86 ± 441.8	0.812
**Echocardiography**			
LVEDD, mm	49.64 ± 2.67	48.67 ± 2.89	0.050
LVESD, mm	36.23 ± 3.32	35.16 ± 3.52	0.078
CO, L/min	4.02 ± 0.88	3.98 ± 0.81	0.770
LVEF, %	50.14 ± 12.37	51.47 ± 13.28	0.557
E, m/s	0.72 ± 0.23	0.81 ± 0.22	0.019
A, m/s	0.73 ± 0.17	0.80 ± 0.18	0.040
E/A	1.00 ± 0.29	1.05 ± 0.31	0.376
e′, m/s	0.08 ± 0.02	0.07 ± 0.03	0.138
E/e′	9.98 ± 3.88	13.26 ± 5.86	< 0.001
**Cardiac function**			0.562
Systolic dysfunction, n (%)	19 (32.76)	18 (24.32)	
Diastolic dysfunction, n (%)	29 (50.00)	42 (56.76)	
Systolic and diastolic dysfunction, n (%)	10 (17.24)	14 (18.92)	
SOFA scores	12.43 ± 1.84	12.41 ± 1.95	0.939
APACHE II scores	24.76 ± 3.21	24.73 ± 3.24	0.959
**CRRT protocol mode**			0.938
CVVH	20 (34.48)	26 (35.14)	
CVVHDF	38 (65.52)	48 (64.86)	
**Anticoagulant**			0.662
Sodium citrate	26 (44.83)	36 (48.65)	
Heparin	32 (55.17)	38 (51.35)	
Blood flow, mL/min	154.14 ± 18.24	151.15 ± 17.72	0.344
CRRT dose, mL/kg/h	26.52 ± 2.10	29.43 ± 3.46	< 0.001
Ultrafiltration dose, mL/kg/h	26.93 ± 2.02	27.22 ± 1.98	0.418
Dialysis dose, mL/kg/h	15.50 ± 1.78	20.00 ± 3.58	< 0.001
**At the start of CRRT**			
Noradrenaline, n (%)	58 (100.00)	44 (59.46)	< 0.001
Noradrenaline, μg/kg/min	0.34 ± 0.20	0.22 ± 0.21	0.003
Six-hour UO before CRRT initiation, mL	391.71 ± 142.64	151.24 ± 60.72	< 0.001
Total duration CRRT, hour	84.83 ± 25.13	85.08 ± 18.79	0.949
Creatinine, mg/dL	1.04 ± 0.31	2.57 ± 0.99	< 0.001
MAP, mmHg	79.28 ± 5.29	89.78 ± 10.78	< 0.001
SOFA	12.74 ± 1.72	13.27 ± 1.72	0.082
APACHE II	27.62 ± 4.14	29.55 ± 4.66	0.014
**At the end of CRRT**			
Noradrenaline, n (%)	23 (39.66)	36 (50.00)	0.291
Noradrenaline, μg/kg/min	0.30 ± 0.14	0.22 ± 0.11	0.013
Creatinine, mg/dL	0.94 ± 0.57	1.83 ± 0.92	< 0.001
MAP, mmHg	96.55 ± 14.24	94.93 ± 15.37	0.536
SOFA	7.98 ± 5.53	9.85 ± 5.85	0.064
APACHE II	17.16 ± 9.05	19.36 ± 9.24	0.171

*sIPTW* stabilized inverse probability of treatment weights, *BMI* body mass index, *MAP* mean arterial pressure, *PLT* platelet, *eGFR* estimated glomerular filtration rate, *BUN* blood urea nitrogen, *CK-MB* creatine kinase-MB, *ALT* alanine aminotransferase, *MV* mechanical ventilation, *LV* left ventricle, *LVEDD* LV end diastolic dimension, *LVESD* LV end systolic dimension, *CO* cardiac output, *LVEF* LV ejection fraction, *CVVH* continuous veno-venous hemofiltration, *CVVHDF* continuous veno-venous hemodiafiltration, *SOFA* Sequential Organ Failure Assessment, *APACHE II* Acute Physiologic Assessment and Chronic Health Evaluation II, *CRRT* continuous renal replacement therapy, *UO* urine output.

At the beginning of CRRT, early CRRT initiated patients had lower MAP (79.28 ± 5.29 mmHg vs. 89.78 ± 10.78 mmHg, *p* < 0.001), a higher proportion of norepinephrine users (100% vs. 59.46%, *p* < 0.001) and were administered higher levels of noradrenaline at the start of CRRT (0.34 ± 0.20 μg/kg/min vs. 0.22 ± 0.21 μg/kg/min, *p* = 0.003). Patients in the control group had worse renal function with higher creatinine (2.57 ± 0.99 mg/dL vs. 1.04 ± 0.31 mg/dL, *p* < 0.001) and lower six-hour urine output (151.24 ± 60.72 mL vs. 391.71 ± 142.64 mL, *p* < 0.001). The APACHE II scores of the control group were higher than the early group (29.55 ± 4.66 vs. 27.62 ± 4.14, *p* = 0.014). The mean duration of CRRT did not differ between the two groups (84.83 ± 25.13 h vs. 85.08 ± 18.79 h, *p* = 0.949). At the end of CRRT, early CRRT initiated patients were administered higher levels of noradrenaline at the start of CRRT (0.30 ± 0.14 μg/kg/min vs. 0.22 ± 0.11 μg/kg/min, p = 0.013). Patients in the control group had worse renal function with higher creatinine (1.83 ± 0.92 mg/dL vs. 0.94 ± 0.57 mg/dL, p < 0.001).

### Patient characteristics after sIPTW between the early group and the control group

After sIPTW, baseline characteristics of the two groups at admission were balanced (Table 2). Patients with early CRRT had worse hemodynamic characteristics when compared to those in the control group at the start of CRRT. Early CRRT-initiated patients had lower MAP (80.39 ± 5.16 mmHg vs. 85.81 ± 10.31 mmHg, *p* = 0.001) and higher proportion of norepinephrine users (100% vs. 74.32%, *p* = 0.001) at the start of CRRT. Patients in the control group had worse renal function with higher creatinine (2.60 ± 1.03 mg/dL vs. 1.01 ± 0.29 mg/dL, *p* < 0.001) and lower six-hour urine output (153.55 ± 57.09 mL vs. 392.02 ± 143.93 mL, *p* < 0.001) at the start of CRRT. The APACHE II (29.85 ± 4.44 vs. 27.43 ± 4.08, *p* = 0.006) scores of the control group were higher than the early group. At the end of CRRT, early CRRT initiated patients had a lower proportion of norepinephrine users (34.04% vs. 62.16%, *p* = 0.008). Patients in the control group had worse renal function with higher creatinine (2.01 ± 0.99 mg/dL vs. 0.93 ± 0.68 mg/dL, *p* < 0.001). The SOFA scores (10.93 ± 5.68 vs. 8.06 ± 5.13, *p* = 0.008) and APACHE II scores of the control group were higher than the early group (21.71 ± 9.41 vs. 17.02 ± 8.13, *p* = 0.007).

**Table 2:** Patient characteristics between early group and control group after sIPTW (at baseline and at the start of CRRT).

Characteristics	Early group (n = 47)	Control group (n = 74)	sIPTW-adjusted *p*
Age, years	63.98 ± 8.98	62.64 ± 8.92	0.443
Male, n (%)	30 (63.83)	54 (72.97)	0.311
BMI, kg/m^2^	24.52 ± 3.32	24.33 ± 2.85	0.746
MAP, mmHg	82.16 ± 5.22	82.64 ± 5.54	0.670
**Laboratory tests**			
Leukocyte count, × 10^9^/L	14.85 ± 3.47	15.03 ± 3.33	0.780
Neutrophil percentage, %	81.55 ± 7.18	80.62 ± 6.68	0.473
PLT count, × 10^9^/L	173.12 ± 56.23	177.14 ± 52	0.702
Blood pH	7.34 ± 0.06	7.34 ± 0.07	0.766
Baseline creatinine, mg/dL	0.92 ± 0.28	1.00 ± 0.32	0.199
Serum potassium, mEq/L	4.77 ± 0.60	4.93 ± 0.66	0.204
Baseline eGFR, mL/min/1.73 m^2^	79.84 ± 22.09	76.80 ± 22.03	0.516
BUN, mg/dL	16.19 ± 2.70	16.12 ± 2.99	0.901
CK-MB, IU/L	23.09 ± 20.31	21.35 ± 18.74	0.685
ALT, U/L	54.16 ± 57.44	69.14 ± 74.81	0.387
Total bilirubin, mg/dL	1.27 ± 0.80	1.39 ± 1.31	0.654
Lactate, mg/dL	66.53 ± 45.63	70.88 ± 50.95	0.727
Hb, g/L	133.20 ± 11.33	131.19 ± 12.66	0.377
Six-hour UO at admission, mL	444.77 ± 153.88	451.62 ± 128.41	0.812
**Primary diagnosis**			
Pneumo-sepsis, n (%)	14 (29.79)	19 (25.68)	0.666
Urosepsis, n (%)	4 (8.51)	4 (5.41)	0.343
Abdominal sepsis, n (%)	14 (29.79)	35 (47.30)	0.098
Other cause, n (%)	15 (31.91)	16 (21.62)	0.356
**Type of surgery**			
Abdominal surgery, n (%)	5 (10.64)	4 (5.41)	0.317
Urinary surgery, n (%)	1 (2.13)	0 (0)	0.088
Others, n (%)	2 (4.26)	1 (1.35)	0.390
Postoperative, n (%)	8 (17.02)	5 (6.76)	0.115
Positive blood culture, n (%)	21 (44.68)	31 (41.89)	0.888
Invasive MV, n (%)	23 (48.94)	51 (68.92)	0.051
**Comorbidities**			
Hypertension, n (%)	14 (29.79)	20 (27.03)	0.806
Diabetes mellitus, n (%)	7 (14.89)	12 (16.22)	0.800
Coronary artery disease, n (%)	3 (6.38)	9 (12.16)	0.347
Heart failure, n (%)	5 (10.64)	8 (10.81)	0.975
**Medication at admission**			
Noradrenaline, n (%)	39 (82.98)	56 (75.68)	0.342
Dopamine, n (%)	13 (27.66)	20 (27.03)	0.942
Glucocorticoid, n (%)	5 (10.64)	17 (22.97)	0.098
Digitalis during hospitalization, n (%)	8 (17.02)	11 (14.86)	0.735
Resuscitation fluid in first 36 h (crystal solution), mL	3094.63 ± 465.57	3045.9 ± 505.00	0.670
**Echocardiography**			
LVEDD, mm	49.19 ± 2.79	49.26 ± 2.80	0.912
LVESD, mm	36.16 ± 3.27	35.94 ± 3.38	0.752
CO, L/min	4.01 ± 0.87	3.95 ± 0.88	0.772
LVEF, %	50.29 ± 12.09	49.48 ± 13.64	0.766
E, m/s	0.76 ± 0.25	0.78 ± 0.23	0.632
A, m/s	0.76 ± 0.18	0.78 ± 0.18	0.656
E/A	1.01 ± 0.29	1.03 ± 0.30	0.713
e′, m/s	0.74 ± 0.22	0.75 ± 0.28	0.872
E/e′	10.76 ± 4.38	11.66 ± 5.29	0.412
**Cardiac function**			0.806
Systolic dysfunction, n (%)	15 (31.91)	25 (33.33)	
Diastolic dysfunction, n (%)	25 (53.19)	34 (45.33)	
Systolic and diastolic dysfunction, n (%)	7 (14.89)	15 (20.00)	
SOFA scores	12.72 ± 1.91	12.67 ± 1.75	0.897
APACHE II scores	24.82 ± 3.24	25.02 ± 3.19	0.776
**CRRT protocol mode**			0.270
CVVH	15 (31.91)	31 (41.89)	
CVVHDF	32 (68.09)	43 (58.11)	
**Anticoagulant**			0.474
Sodium citrate	21 (44.68)	38 (51.35)	
Heparin	26 (55.32)	36 (48.65)	
Blood flow, mL/min	154.87 ± 17.32	151.6 ± 17.58	0.333
CRRT dose, mL/kg/h	26.96 ± 2.08	27.96 ± 3.45	0.077
Ultrafiltration dose, mL/kg/h	26.98 ± 2.00	27.59 ± 2.09	0.178
Dialysis dose, mL/kg/h	15.41 ± 1.59	20.84 ± 3.78	< 0.001
**At the start of CRRT**			
Noradrenaline, n (%)	47 (100)	55 (74.32)	0.001
Noradrenaline, μg/kg/min	0.31 ± 0.19	0.27 ± 0.22	0.418
Six-hour UO before CRRT initiation, mL	392.02 ± 143.93	153.55 ± 57.09	< 0.001
Total duration CRRT, hour	78.58 ± 29.15	83.45 ± 18.54	0.463
Creatinine, mg/dL	1.01 ± 0.29	2.60 ± 1.03	< 0.001
MAP, mmHg	80.39 ± 5.16	85.81 ± 10.31	0.001
SOFA	13.76 ± 1.79	14.22 ± 2.05	0.191
APACHE II	27.43 ± 4.08	29.85 ± 4.44	0.006
**At the end of CRRT**			
Noradrenaline, n (%)	16 (34.04)	46 (62.16)	0.008
Noradrenaline, μg/kg/min	0.31 ± 0.13	0.23 ± 0.11	0.065
Creatinine, mg/dL	0.93 ± 0.68	2.01 ± 0.99	< 0.001
MAP, mmHg	97.18 ± 15.34	91.79 ± 13.91	0.069
SOFA	8.06 ± 5.13	10.93 ± 5.68	0.008
APACHE II	17.02 ± 8.13	21.71 ± 9.41	0.007

*sIPTW* stabilized inverse probability of treatment weights, *BMI* body mass index, *MAP* mean arterial pressure, *PLT* platelet, *eGFR* estimated glomerular filtration rate, *BUN* blood urea nitrogen, *CK-MB* creatine kinase-MB, *ALT* alanine aminotransferase, *MV* mechanical ventilation, *LV* left ventricle, *LVEDD* LV end diastolic dimension, *LVESD* LV end systolic dimension, *CO* cardiac output, *LVEF* LV ejection fraction, *CVVH* continuous veno-venous hemofiltration, *CVVHDF* continuous veno-venous hemodiafiltration, *SOFA* Sequential Organ Failure Assessment, *APACHE II* Acute Physiologic Assessment and Chronic Health Evaluation II, *CRRT* continuous renal replacement therapy, *UO* urine output.

### Early CRRT was associated with a lower ICU mortality

Before sIPTW, the ICU mortality of patients receiving early CRRT was significantly lower than that in the control group (32.8% vs. 52.7%, *p* = 0.022). The invasive mechanical ventilation (MV) and vasoactive agent initiation in the early group were shorter than those in the control group (10.66 ± 5.49 days vs. 14.38 ± 4.98 days, *p* = 0.002 and 4.12 ± 2.08 days vs. 7.86 ± 2.64 days, *p* < 0.001). After sIPTW, the ICU mortality was still significantly different in the early group versus the control group (27.7% versus 63.5%, *p* < 0.001). The length of invasive MV and vasoactive agent initiation (9.95 ± 5.42 days vs. 12.24 ± 4.77 days, *p* = 0.003 and 4.16 ± 1.97 days vs. 8.11 ± 2.70 days, *p* < 0.001) were significantly different between two groups (Table 3).

**Table 3:** Clinical outcomes between early group and control group before and after sIPTW.

Outcome	Early group	Control group	*P*
**Pre-adjusted**			
Death, n (%)	19 (32.76)	39 (52.70)	0.022
ICU stay, days	19.90 ± 6.55	21.12 ± 7.93	0.344
Invasive MV, days	10.66 ± 5.49	14.38 ± 4.98	0.002
Vasoactive agent, days	4.12 ± 2.08	7.86 ± 2.64	< 0.001
**sIPTW-adjusted**			
Death, n (%)	13 (27.66)	47 (63.51)	< 0.001
ICU stay, days	19.08 ± 5.42	21.67 ± 8.11	0.133
Invasive MV, days	9.95 ± 5.42	12.24 ± 4.77	0.003
Vasoactive agent, days	4.16 ± 1.97	8.11 ± 2.70	< 0.001

*sIPTW* stabilized inverse probability of treatment weights, *ICU* intensive care unit, *MV* mechanical ventilation.

### Early CRRT was associated with a lower risk of ICU death

The weighted univariate logistic regression analysis showed that early CRRT initiation was a protective factor and was associated with a lower risk of ICU death compared with the control group (OR 0.208; 95% CI 0.093–0.464; *p* < 0.001; Table 4). By weighted multivariable analysis (Table 5), early CRRT initiation was a protective factor for the risk of ICU mortality when the variables were screened using a step-by-step method, and early CRRT was associated with a lower risk of ICU death compared with the control group (OR 0.149; 95% CI 0.051–0.434; *p* < 0.001). Meanwhile, MAP was a protective factor for the risk of ICU mortality (OR 0.924; 95% CI 0.870–0.982; *p* = 0.011). In addition, the risk factors associated with ICU mortality included abdominal sepsis (OR 3.150; 95% CI 1.076–9.223; *p* = 0.036), and invasive MV (OR 17.841; 95% CI 5.524–57.621; *p* < 0.001).

**Table 4:** Weighted univariate logistic regression analysis.

Characteristic	OR	95% CI	*P*
Age, years	0.955	0.915, 0.997	0.037
Gender (Female vs. Male)	2.010	0.909, 4.445	0.085
BMI, kg/m^2^	0.978	0.869, 1.101	0.711
MAP, mmHg	1.012	0.947, 1.081	0.728
**Laboratory tests**			
Leukocyte count, × 10^9^/L	0.926	0.831, 1.033	0.167
Neutrophil percentage, %	0.957	0.907, 1.010	0.112
PLT count, × 10^9^/L	0.996	0.990, 1.003	0.279
Blood pH, per 0.1	0.929	0.545, 1.584	0.788
Baseline Creatinine, mg/dL	2.728	0.837, 8.891	0.096
Serum potassium, mEq/L	1.744	0.967, 3.146	0.065
Baseline eGFR, mL/min/1.73 m^2^	0.997	0.981, 1.013	0.705
BUN, mg/dL	1.019	0.899, 1.154	0.772
CK-MB, IU/L	0.999	0.981, 1.018	0.941
ALT, U/L	1.007	1.001, 1.013	0.028
Total Bilirubin, mg/dL	1.297	0.923, 1.821	0.134
Lactate, mg/dL	1.007	0.999, 1.016	0.084
Hb, g/L	0.989	0.961, 1.019	0.482
Six-hour UO at admission, mL	0.999	0.997, 1.002	0.545
**Primary diagnosis**			
Pneumo-sepsis	0.809	0.363, 1.801	0.603
Urosepsis	0.500	0.108, 2.314	0.375
Abdominal sepsis	2.700	1.274, 5.721	0.010
Other cause	0.443	0.190, 1.033	0.059
**Type of surgery**			
Abdominal surgery, n (%)	0.809	0.198, 3.298	0.767
Urinary surgery, n (%)	1.168	0.020, 66.791	0.940
Others, n (%)	0.372	0.032, 4.330	0.430
Postoperative	0.673	0.207, 2.193	0.511
Positive blood culture	1.608	0.778, 3.323	0.200
Invasive MV	13.615	5.315, 34.874	< 0.000
**Comorbidities**			
Hypertension	1.137	0.513, 2.524	0.751
Diabetes mellitus	1.972	0.704, 5.529	0.197
Coronary artery disease	7.729	1.388, 43.031	0.020
Heart failure	1.521	0.472, 4.904	0.483
**Medication at admission**			
Noradrenaline	1.172	0.494, 2.780	0.720
Dopamine	2.189	0.960, 4.993	0.063
Glucocorticoid	1.642	0.633, 4.258	0.308
Digitalis during hospitalization, n (%)	0.827	0.309, 2.216	0.705
Resuscitation fluid in first 36 h (crystal solution), mL	1.000	0.999, 1.000	0.520
**Echocardiography**			
LVEDD, mm	1.160	1.011, 1.331	0.035
LVESD, mm	1.082	0.969, 1.207	0.162
CO, L/min	0.973	0.645, 1.469	0.898
LVEF, %	0.986	0.959, 1.014	0.323
E, m/s	0.476	0.102, 2.225	0.345
A, m/s	0.138	0.017, 1.096	0.061
E/A	1.700	0.496, 5.820	0.398
e′, per 0.1 m/s	1.936	0.485, 7.722	0.349
E/e′	0.957	0.889, 1.031	0.248
**Cardiac function**			
Systolic dysfunction	Ref.		
Diastolic dysfunction	0.657	0.292, 1.477	0.310
Combined systolic and diastolic dysfunction	0.784	0.275, 2.234	0.649
**CRRT protocol mode**			
CVVH	Ref.		
CVVHDF	0.520	0.247, 1.095	0.085
**Anticoagulant**			
Sodium citrate	Ref.		
Heparin	0.481	0.233, 0.994	0.048
Blood flow, mL/min	0.997	0.977, 1.018	0.803
CRRT dose, mL/kg/h	0.856	0.753, 0.973	0.017
Ultrafiltration dose, mL/kg/h	1.117	0.938, 1.331	0.213
Dialysis dose, mL/kg/h	1.150	1.020, 1.296	0.022
SOFA scores	1.124	0.919, 1.373	0.255
APACHE II scores	1.053	0.940, 1.179	0.371
**At the start of CRRT**			
Noradrenaline	1.416	0.528, 3.800	0.490
Noradrenaline, per 0.1 μg/kg/min	1.172	0.963, 1.426	0.112
Six-hour UO before CRRT initiation, mL	0.997	0.994, 0.999	0.012
Total duration CRRT, hour	0.999	0.984, 1.015	0.918
Creatinine, mg/dL	1.465	1.046, 2.051	0.026
MAP, mmHg	0.954	0.913, 0.997	0.038
SOFA	1.130	0.938, 1.361	0.198
APACHE II	1.084	0.998, 1.178	0.057
**At the end of CRRT**			
Noradrenaline, n (%)	252.215	54.624, 1164.566	< 0.001
Noradrenaline, μg/kg/min	2.219	0.708, 6.957	0.172
Creatinine, mg/dL	35.411	10.279, 121.993	< 0.001
MAP, mmHg	0.817	0.756, 0.883	< 0.001
SOFA	2.518	1.685, 3.762	< 0.001
APACHE II	1.462	1.277, 1.673	< 0.001
Early CRRT (vs. Control)	0.208	0.093, 0.464	< 0.001

*BMI* body mass index, *MAP* mean arterial pressure, *PLT* platelet, *eGFR* estimated glomerular filtration rate, *BUN* blood urea nitrogen, *CK-MB* creatine kinase-MB, *ALT* alanine aminotransferase, *MV* mechanical ventilation, *LV* left ventricle, *LVEDD* LV end diastolic dimension, *LVESD* LV end systolic dimension, *CO* cardiac output, *LVEF* LV ejection fraction, *SOFA* Sequential Organ Failure Assessment, *APACHE II* Acute Physiologic Assessment and Chronic Health Evaluation II, *CRRT* continuous renal replacement therapy, *UO* urine output, *OR* odds ratio, *CI* confidence interval.

**Table 5:** Weighted multivariable logistic regression analysis.

Characteristic	OR	95% CI	*p*
Early CRRT (vs. control)	0.149	0.051, 0.434	< 0.001
Abdominal sepsis	3.150	1.076, 9.223	0.036
Invasive MV	17.841	5.524, 57.621	< 0.001
MAP at the start of CRRT	0.924	0.870, 0.982	0.011

*CRRT* continuous renal replacement therapy, *MV* mechanical ventilation, *OR* odds ratio, *CI* confidence interval.

### Subgroup analyses

Patients with AKI in the control group received either an accelerated strategy for CRRT initiation (40, 54.1%) or a standard strategy (37, 45.9%). The characteristics of patients at the time of CRRT and features of the initial prescription are provided in Tables 6 and 7. CRRT was initiated at 7.16 ± 2.91 h in the accelerated-strategy group, and 39.03 ± 19.26 h in the standard-strategy group (*p* < 0.001). The proportion of CRRT protocol mode was significantly different between the two groups (*p* = 0.016). No significant difference between two groups was observed regarding ICU mortality (52.5% vs. 52.9%, *p* = 0.970). The invasive MV days of accelerated-strategy group was fewer than standard-strategy group (12.57 ± 4.61 days vs. 16.13 ± 4.78 days, *p* = 0.013).

**Table 6:** Patient characteristics between accelerated-strategy group and standard-strategy group (at baseline and at the start of CRRT).

Characteristics	Accelerated strategy (n = 40)	Standard strategy (n = 34)	*p*
Age, years	61.48 ± 9.14	63.88 ± 8.50	0.248
Male, n (%)	27 (67.50)	23 (67.65)	0.989
BMI, kg/m^2^	24.74 ± 3.12	24.78 ± 3.33	0.965
MAP, mmHg	83.53 ± 5.06	82.91 ± 5.69	0.625
**Laboratory tests**			
Leukocyte count, × 10^9^/L	15.75 ± 3.28	14.9 ± 3.47	0.286
Neutrophil percentage, %	81.05 ± 6.11	80.80 ± 8.40	0.888
PLT count, × 10^9^/L	175.92 ± 57.34	179.82 ± 50.88	0.760
Blood pH	7.33 ± 0.06	7.35 ± 0.06	0.060
Baseline creatinine, mg/dL	0.89 ± 0.28	1.00 ± 0.31	0.102
Serum potassium, mEq/L	4.89 ± 0.69	4.79 ± 0.68	0.538
Baseline eGFR, mL/min/1.73 m^2^	84.32 ± 20.14	75.02 ± 23.40	0.070
BUN, mg/dL	16.20 ± 2.77	15.57 ± 3.51	0.407
CK-MB, IU/L	20.15 ± 16.15	22.26 ± 13.21	0.544
ALT, U/L	59.63 ± 57.42	45.29 ± 42.96	0.235
Total bilirubin, mg/dL	1.31 ± 1.17	1.08 ± 0.71	0.319
Lactate, mg/dL	58.44 ± 27.89	59 ± 37.78	0.941
Hb, g/L	131.78 ± 14.46	130.50 ± 10.60	0.671
Six-hour UO at admission, mL	461.53 ± 150.72	459.29 ± 126.9	0.946
**Primary diagnosis**			
Pneumo-sepsis, n (%)	9 (22.50)	14 (41.18)	0.084
Urosepsis, n (%)	1 (2.50)	4 (11.76)	0.173
Abdominal sepsis, n (%)	18 (45.00)	10 (29.41)	0.168
Other cause, n (%)	12 (30.00)	6 (17.65)	0.217
**Type of surgery**			
Abdominal surgery, n (%)	2 (5.00)	1 (2.94)	> 0.999
Urinary surgery, n (%)	0 (0)	0 (0)	–
Others, n (%)	0 (0)	1 (2.94)	0.459
Postoperative, n (%)	2 (5.00)	2 (5.88)	> 0.999
Positive blood culture, n (%)	13 (32.50)	17 (50.00)	0.127
Invasive MV, n (%)	23 (57.50)	24 (70.59)	0.244
**Comorbidities**			
Hypertension, n (%)	7 (17.50)	12 (35.29)	0.081
Diabetes mellitus, n (%)	6 (15.00)	6 (17.65)	0.758
Coronary artery disease, n (%)	3 (7.50)	2 (5.88)	> 0.999
Heart failure, n (%)	2 (5.00)	7 (20.59)	0.071
**Medication at admission**			
Noradrenaline, n (%)	27 (67.50)	22 (64.71)	0.800
Dopamine, n (%)	6 (15.00)	6 (17.65)	0.758
Glucocorticoid, n (%)	7 (17.50)	6 (17.65)	0.987
Digitalis during hospitalization, n (%)	6 (15.00)	5 (14.71)	0.972
Resuscitation fluid in first 36 h (crystal solution), mL	3066.25 ± 520.90	3063.24 ± 333.33	0.976
**Echocardiography**			
LVEDD, mm	48.82 ± 3.01	48.49 ± 2.77	0.629
LVESD, mm	35.18 ± 3.53	35.14 ± 3.55	0.965
CO, L/min	3.93 ± 0.88	4.05 ± 0.72	0.522
LVEF, %	51.06 ± 14.33	51.95 ± 12.13	0.774
E, m/s	0.80 ± 0.23	0.83 ± 0.21	0.665
A, m/s	0.83 ± 0.19	0.76 ± 0.16	0.138
E/A	0.99 ± 0.27	1.11 ± 0.34	0.115
e′, m/s	0.07 ± 0.03	0.07 ± 0.03	0.879
E/e′	13.11 ± 6.12	13.44 ± 5.63	0.814
**Cardiac function**			0.778
Systolic dysfunction, n (%)	11 (27.50)	7 (20.59)	
Diastolic dysfunction, n (%)	22 (55.00)	20 (58.82)	
Systolic and diastolic dysfunction, n (%)	7 (17.50)	7 (20.59)	
SOFA scores	12.65 ± 2.07	12.12 ± 1.79	0.245
APACHE II scores	25.38 ± 3.36	23.97 ± 2.98	0.063
**CRRT protocol mode**			0.016
CVVH	19 (47.50)	7 (20.59)	
CVVHDF	21 (52.50)	27 (79.41)	
**Anticoagulant**			0.098
Sodium citrate	23 (57.50)	13 (38.24)	
Heparin	17 (42.50)	21 (61.76)	
Blood flow, mL/min	154.25 ± 18.69	147.5 ± 16.01	0.103
CRRT dose, mL/kg/h	29.35 ± 3.43	29.53 ± 3.55	0.826
Ultrafiltration dose, mL/kg/h	27.33 ± 2.03	27.09 ± 1.94	0.612
Dialysis dose, mL/kg/h	20.48 ± 4	19.63 ± 3.25	0.422
**At the start of CRRT**			
Noradrenaline, n (%)	21 (100)	23 (100)	–
Noradrenaline, μg/kg/min	0.24 ± 0.23	0.2 ± 0.19	0.524
Six-hour UO before CRRT initiation, mL	142.55 ± 65.46	161.47 ± 53.78	0.183
Total duration CRRT, hour	84.35 ± 21.93	85.94 ± 14.54	0.711
Creatinine, mg/dL	2.59 ± 1.06	2.55 ± 0.91	0.871
MAP, mmHg	90.35 ± 10.47	89.12 ± 11.25	0.627
SOFA	14.38 ± 2.24	13.76 ± 2.12	0.235
APACHE II	30.35 ± 4.62	28.62 ± 4.61	0.112
**At the end of CRRT**			
Noradrenaline, n (%)	21 (52.50)	16 (47.06)	0.641
Noradrenaline, μg/kg/min	0.23 ± 0.12	0.20 ± 0.11	0.512
Creatinine, mg/dL	1.92 ± 1.01	1.72 ± 0.82	0.365
MAP, mmHg	96.78 ± 17.59	92.76 ± 12.17	0.253
SOFA	9.83 ± 5.93	9.88 ± 5.85	0.967
APACHE II	19.73 ± 9.63	18.94 ± 8.89	0.719
CRRT start time, hours	7.16 ± 2.91	39.03 ± 19.26	< 0.001

*BMI* body mass index, *MAP* mean arterial pressure, *PLT* platelet, *eGFR* estimated glomerular filtration rate, *BUN* blood urea nitrogen, *CK-MB* creatine kinase-MB, *ALT* alanine aminotransferase, *MV* mechanical ventilation, *LV* left ventricle, *LVEDD* LV end diastolic dimension, *LVESD* LV end systolic dimension, *CO* cardiac output, *LVEF* LV ejection fraction, *SOFA* Sequential Organ Failure Assessment, *APACHE II* Acute Physiologic Assessment and Chronic Health Evaluation II, *CRRT* continuous renal replacement therapy, *UO* urine output, *OR* odds ratio, *CI* confidence interval.

**Table 7:** Clinical outcomes between accelerated-strategy group and standard-strategy group.

Outcome	Accelerated Strategy	Standard Strategy	*p*
Death, n (%)	21 (52.50)	18 (52.94)	0.970
ICU stay, days	21.23 ± 8.51	21.00 ± 7.32	0.904
Invasive MV, days	12.57 ± 4.61	16.13 ± 4.78	0.013
Vasoactive agent, days	7.76 ± 2.31	7.96 ± 2.97	0.774

*sIPTW* stabilized inverse probability of treatment weights, *ICU* intensive care unit, *MV* mechanical ventilation.

## Discussion

In this multicenter retrospective study, for hemodynamically unstable sepsis patients with LV dysfunction, the ICU mortality was lower, the invasive MV days and vasoactive agent initiation days were fewer in those receiving CRRT with the absence of AKI compared with those who accepted CRRT following AKI. However, accelerated strategy of CRRT initiation was not associated with a survival benefit for patients with AKI, although a benefit of fewer invasive MV days was detected. Our findings provide clues for the treatment strategy of hemodynamically unstable sepsis with LV dysfunction, which can easily develop to organ perfusion including kidney injury.

CRRT can be life-saving by correcting metabolic disorders in patients with severe acidosis and hyperkalemia, stabilizing hemodynamics, controlling disturbances of fluid metabolism in patients with severe pulmonary edema, and removing toxins and circulating inflammatory cytokines in patients with severe sepsis^15^. Our study showed that the creatinine level of two groups were lower than that before the initiation of CRRT, and have significant difference at the end of CRRT between two groups. The all-cause mortality of severe sepsis and septic shock patients with LV dysfunction and initiated CRRT enrolled in our study was 43.9%, which was similar to the 28-day all-cause mortality (43.1%) of LV systolic asynchrony in patients with septic shock^16^. However, the mortality of all patients receiving early CRRT was 32.8%, which was lower significantly and also appeared better than the recently reported data on septic patients with AKI treated with CRRT (62.0%)^17^. ICU mortality in the early group was slightly lower than the 28-day mortality in the STARRT AKI trials^18^. We speculate that the different CRRT startup time, which is before the AKI, and the different disease types may cause such difference. The prognosis of CRRT for AKI induced by different causes may be different^15^. In our follow-up subgroup study, ICU mortalities in the accelerated- and standard-strategy group were higher. Some of our patients died in hospital after more than 28 days.

Sepsis-related LV dysfunction, known as septic cardiomyopathy, was observed in nearly 48% of the severe sepsis and septic shock patients^19^. In the present study, the number of patients with sepsis-related LV dysfunction was 116 (87.9%), and only 16 (12.1%) patients had pre-existing heart failure (HF). Regarding the treatment of septic cardiomyopathy, there have been no specific therapeutics so far. The current guidelines for the management of septic shock, for example infection control with adequate antibiotics and hemodynamic stabilization with inotropic and vasopressor agents and fluids, represent the cornerstone of septic cardiomyopathy therapy. Innovative therapeutic management strategies of septic cardiomyopathy are therefore urgently needed^3,20^. It is noteworthy that those treated with early CRRT had worse hemodynamics than those receiving delayed CRRT, as reflected by a higher proportion of norepinephrine users, higher noradrenaline levels, and lower MAP at the initiation of treatment. MAP, the main measurement for dynamic instability, is a key determinant of mean systemic filling pressure driving cardiac output (CO) and venous return. Increasing MAP therefore usually results in increased tissue blood flow and augments the supply side of tissue perfusion. Some tissues such as the brain and kidneys can auto-regulate blood flow. MAPs below a threshold, usually 60 mmHg, are associated with decreased organ perfusion, which tracks linearly with MAP^1^. A randomized controlled trial (RCT) demonstrated a 10.5% absolute reduction in mortality in RRT with higher MAP targets among chronic hypertension patients^21^. The panel of IDEALICU Trial recommended RRT in patients with sepsis, AKI, and there are other absolute dialysis indications including refractory fluid overload^22^. In our study, the vasoactive drug dependence time and invasive MV time were lower in the early group. The MAP was significantly higher and the noradrenaline use was less after CRRT in the early group. In addition, MAP was a protective factor for the risk of ICU mortality. We speculate that the early intervention of CRRT may improve MAP and hemodynamics, and subsequently the cardiac function and attenuate pulmonary edema. The critical severity scores including SOFA and APACHE II were lower after CRRT. Although the APACHE II scores of the control group were higher than the early group at the start of CRRT, it was not associated with a higher risk of ICU death after weighted logistic regression analysis. A recent meta-analysis also supports this possible benefit of early RRT initiation as shown by fewer MV days^23^.

The pathophysiological interplay between the heart and kidney was defined as cardiorenal syndrome (CRS), which has been associated with all-cause mortality in patients with sepsis^5,6,24–27^. In the current study, type 5 and type 1 CRS were witnessed in the two subgroups. Systemic diseases, especially sepsis, are the most common causes of type 5 CRS^28^, which was detected in 67–76% of the septic population and was an independent predictor of in-hospital mortality^29^. Cardiovascular dysfunction in septic CRS-5 can manifest as septic cardiomyopathy, circulatory failure, and autonomic dysregulation^28^. Septic cardiomyopathy is a fundamental feature of sepsis-associated cardiac dysfunction^3^ that includes LVSD and LVDD, contributing to renal hypoperfusion^30,31^. Type 1 CRS in sepsis patients was represented by decreased LVEF and cardiac output. Elevated central venous pressure increases “kidney afterload” and leads to renal dysfunction, which plays a major role in the pathophysiology of CRS in acute cardiac dysfunction^27^. Other contributing factors are the activation of neurohormonal pathways and proinflammatory responses^32^. Currently, there is no consensus regarding early vs. late CRRT initiation in patients with septic CRS. In our subgroup study, an accelerated CRRT was not associated with benefit clinical outcomes. These findings were consistent with recent studies^15,18,23,33^. The noradrenaline use rate and the creatinine level at the end of CRRT were not different between two subgroups. We considered that once cardiac dysfunction develops into CRS, organ perfusion may further worsen, and CRRT initiation would not provide a survival benefit. Therefore, the duration of dependence on vasoactive agent initiation was longer, and the mortality was higher in the control group compared with the early group. The outcomes were not superior in the accelerated-strategy group. The physiological benefits of ultrafiltration include the removal of inflammatory mediators and precise targeting of fluid removal^27^. The pre-existing LV dysfunction may abruptly worsen, resulting in renal hypoperfusion through a reduction in blood flow or an increase in central venous pressure, eventually leading to type 1 CRS^5^. Ultrafiltration had beneficial effects on hemodynamic changes, which might improve kidney function by reducing renal venous pressure and optimizing renal perfusion^27,34^. In HF patients, CRRT had positive effects on hemodynamics by improving myocardial performance, measured by increased stroke volume, cardiac output and cardiac cycle efficiency^12,34^. Therefore, the accurate volume control and achievement of hemodynamic stability is extremely important and should be carried out as early as possible after detection of a myocardial dysfunction in a patient with sepsis.

Septic cardiomyopathy is primarily caused by the release of inflammatory cytokines, including interleukin-1 beta (IL-1β), IL-6, and tumor necrosis factor-alpha (TNF-α), in addition to tissue hypoxia and mitochondrial dysfunction that leads to cardiac myocyte injury^19,35–38^. Administration of endotoxin in healthy volunteers results in an increase in LV end diastolic volume and a reduction in LVEF^38^. The improvement of myocardial suppression by CRRT accelerates the recovery of cardiac function and improves hemodynamics. AN69 membranes or RENAFLO hemofilters were used in our study, which combined hemoperfusion in some septic shock patients. Polyacrylonitrile (AN69) filter membranes adsorb cytokines during CVVH. The CRRT protocol modes between two groups were not different. The dialysis dose of control group was high for the worse renal function and higher blood potassium levels. The Oxiris-AN69 membrane, HA380 cytokine hemoadsorption and CytoSorb have been examined in many small-scale study series or are under evaluation as measures to improve clinical outcomes in septic shock^39,40^. However, the sample size is small. Standard CRRT was performed in the current study and we did not investigate whether high cut-off membrane therapy, combined hemoperfusion or these filters could increase the clearance of cytokines, such as TNF-α and IL-10. There is insufficient evidence to recommend other blood purification techniques^1^.

We found that early CRRT was associated with a lower risk of ICU death even after weighted multivariable analysis. In addition, abdominal sepsis and invasive MV were risk factors associated with ICU mortality. We suggest that hemodynamically unstable patients with severe sepsis and septic shock complicated with LV dysfunction should be treated with CRRT before the onset of AKI, since hemodynamic stability and clearance of endotoxin is likely to improve cardiac function and survival rates.

This study has several limitations. Firstly, the retrospective nature of the study limited establishing causal relationships and the number of cases is relatively low. Secondly, due to the retrospective nature of the study, it was impossible to carry out continuous monitoring and follow-up of LV function in patients with persistent cardiac dysfunction, which may influence the primary outcome. Thirdly, other mechanisms of action including mitochondrial dysfunction, nitric oxide and danger-associated molecular patterns (DAMPs) are closely linked to sepsis-induced myocardial dysfunction and prognosis^3^. Whether CRRT is effective for the treatment of all these pathological reactions remains unknown. The criteria for the initiation of CRRT, the definition of AKI and CRRT modalities greatly varied in previous studies^15^. Hence, future animal experiments and RCTs are necessary to confirm our results.

## Conclusions

For hemodynamically unstable patients with severe sepsis and septic shock combined with LV dysfunction, an early CRRT performed before the presence of AKI is associated with a lower ICU all-cause mortality.

## Methods

### Study patients and design

This multicenter retrospective study was performed using data from three ICUs located at Fujian Medical University Union Hospital and Fujian Provincial Hospital with a total of 85 beds from January 1, 2013 to December 31, 2019. All participants underwent transthoracic echocardiography within 24 h of admission to identify the presence or absence of LV dysfunction. The exclusion criteria included: younger than 18 years of age, moderate-to-severe valvular heart disease, history of end-stage renal disease or hemodialysis, postrenal causes of renal injury, cardiopulmonary resuscitation before ICU admission, intoxication, cirrhosis, rhabdomyolysis, active malignancy, connective tissue diseases, pregnancy, expected survival less than 24 h, normal LV function, poor echocardiographic image quality.

All patients included in this study were managed with CRRT. Some hemodynamically unstable patients receiving CRRT did not have septic AKI before CRRT. Patients were divided into one of two groups according to the baseline AKI status: the early group (no AKI) or the control group (with AKI). In the early group, early initiation of CRRT was performed in the absence of AKI, though AKI could occur thereafter. The control group received CRRT when AKI was presented. Then, the patients of control group were divided to subgroups that receive an accelerated strategy of CRRT (therapy was initiated within 12 h after the patient had met the eligibility criteria) or a standard strategy (therapy was initiated after conventional indications developed or AKI persisted for > 72 h)^18^. Clinical outcomes included all-cause ICU mortality, length of ICU stay, invasive MV days and vasoactive agent days.

### Data collection

Data concerning demographic and clinical information (primary diagnosis and baseline comorbidities at admission), physiological parameters (hemodynamic data, vasoactive medications and inotropic agents), transthoracic echocardiographic parameters, laboratory results, and the use of invasive MV were extracted from electronic medical records by trained medical staff. Information of the CRRT was reviewed. The urine output (UO) (six-hour UO after admission and before CRRT) and serum creatinine levels (baseline level, maximum during ICU stay, at the start of CRRT and at the end of CRRT) were obtained to verify the presence of AKI. The corresponding glomerular filtration rate (eGFR) was calculated using the Modification of Diet in Renal Disease (MDRD) equation^41^. Baseline disease severity was assessed by the Sequential Organ Failure Assessment (SOFA) score and Acute Physiology and Chronic Health Evaluation (APACHE) II score.

### Definitions: severe sepsis, septic shock, and septic AKI

Severe sepsis was defined as sepsis related to organ dysfunction, hypoperfusion, or hypotension^19^. A lactate level ≥ 2.3 mmol/L (22.1 mg/dL) was considered indicative of hypoperfusion. Hypotension was defined as systolic blood pressure ≤ 90 mmHg or a decrease of 40 mmHg below baseline, organ dysfunction as SOFA score ≥ 2^19^. Septic shock was defined as sepsis-induced persistent hypotension requiring vasopressor therapy to maintain a mean arterial pressure (MAP) of ≥ 65 mmHg or a lactate level ≥ 2.3 mmol/L (22.1 mg/dL) after adequate fluid resuscitation^1,42^. The “septic shock” definition was from the “sepsis-3.0”^1,42^, and “severe sepsis” was cited from the 2001 definition^19^ which had not changed. Septic AKI was defined as the simultaneous presence of sepsis criteria^42^ and the consensus criteria for AKI according to the Kidney Disease: Improving Global Outcomes (KDIGO) guidelines^43^. The baseline creatinine value was either obtained from clinical files within seven to 365 days previous to admission or the minimum inpatient values during the first 7 days of admission^6^. AKI was defined as meeting one of the following criteria: an increase in creatinine by ≥ 0.3 mg/dL within 48 h; an increase in creatinine to ≥ 1.5 times baseline within the previous 7 days; or urine output ≤ 0.5 mL/kg/h for 6 h.

### Transthoracic echocardiographic examination

All echocardiograms were assessed by a professional cardiologist. Two-dimensional, M-mode, and Doppler data were used to obtain parameters from parasternal long- and short-axis views; apical four-chamber, two-chamber, and long-axis views; and subcostal views. Data on early diastolic velocity of mitral inflow (E), early diastolic mitral annular velocity (e′), late diastolic velocity of mitral inflow (A), E/e′ ratio, and E/A ratio were collected. According to the American Society of Echocardiography 2009 guidelines^44^ and the simplified definition suggested by Lanspa et al.^45^, LVSD was defined as LV ejection fraction (LVEF) < 50% (by M-mode sonography), and LV diastolic function was classified into four grades (normal and grades I, II, and III).

### CRRT settings

CRRT was performed in either continuous veno-venous hemofiltration (CVVH) or continuous veno-venous hemodiafiltration (CVVHDF) through the femoral or internal jugular veins at the discretion of attending physicians. PRISMAFLEX and AQUARIUS hemofiltration systems were used with the addition of bicarbonate or potassium if necessary. The dialysate rate, replacement fluid rate, and ultrafiltration rate were adjusted according to patients’ diagnoses, hemodynamic parameters, and fluid overload. AN69 membranes or RENAFLO hemofilters were used and blood flow rates were kept between 100 and 200 mL/min during the procedure. CRRT dose was quantified by effluent rate normalized to body weight (unit: mL/kg/h) and prescribed in the range of 25–35 mL/kg/h.

### Statistical analyses

Continuous variables were expressed as the mean ± standard deviation for normally distributed data and differences between groups were determined using a two independent samples t-test. Data without normal distribution were expressed as the median (interquartile range, P25, and P75) and two groups were compared using the Mann–Whitney *U* test. Categorical variables were presented as counts (percentages) and compared using Pearson’s chi-square test or Fisher’s exact test.

Propensity score weighting (PSW) was applied to balance the baseline characteristics between groups. Firstly, the baseline characteristics were compared between the two groups and the subgroups. Secondly, logistic regression analysis was used to evaluate the probability of treatment with early CRRT or not. With the treatment allocation as dependent variables, and the factors with *p* values < 0.10 between the two groups at admission were taken as the candidate independent variables. The logistic regression model was constructed to calculate the individual propensity score. Thirdly, patients were weighted by the stabilized inverse probability of treatment weighting (sIPTW) and the weighted baseline characteristics were tested again. Clinical outcomes were compared between two groups by chi square analysis and an independent samples *t* test before and after weighting.

The risk factors associated with ICU mortality were further analyzed, and the impact of early CRRT on mortality was evaluated. With mortality as the dependent variable, and baseline clinical characteristics at admission, at the start of CRRT and at the end of CRRT as independent variables, the weighted univariate logistic regression analyses were conducted separately. According to the results of univariate regression analysis, the factors with *p* < 0.05 were selected as the candidate independent variable to construct the multivariate weighted logistic regression model. The step-by-step method was used to screen the variables. Effect size was presented as the odds ratio (OR) with the corresponding 95% confidence interval (CI). The above methods were also applied to the two subgroups. All data were analyzed using R 4.0.2 software, and *p* < 0.05 was considered statistically significant.

### Ethics approval and consent to participate

This study was conducted in accordance with the Declaration of Helsinki and was approved by the research ethics committee of Fujian Medical University Union Hospital (Ethics Code: 2019KJCX006) and Fujian Provincial Hospital (Ethics Code: K2020-05-014). Informed consent was waived by Fujian Medical University Union Hospital Ethics Committee and Fujian Provincial Hospital Ethics Committee due to the retrospective and observational nature of the study.

## Supplementary Information

Supplementary Table S1.

Supplementary Information 1.

Supplementary Information 2.

## Data Availability

All data generated or analyzed during this study are included in this published article [and its supplementary information files].

## Abbreviations

LV: Left ventricular
AKI: Acute kidney injury
LVDD: Left ventricular diastolic dysfunction
LVSD: Left ventricular systolic dysfunction
CRRT: Continuous renal replacement treatment
RRT: Renal replacement treatment
ICU: Intensive care unit
UO: Urine output
eGFR: Estimated glomerular filtration rate
APACHE II: Acute physiology and chronic health evaluation II
SOFA: Sequential organ failure assessment
MAP: Mean arterial pressure
KDIGO: Kidney Disease: Improving Global Outcomes
BMI: Body mass index
PLT: Platelet
BUN: Blood urea nitrogen
ALT: Alanine aminotransferase
CK-MB: Creatine kinase-MB
LVEDD: Left ventricular end-diastolic diameter
LVESD: Left ventricular end-systolic diameter
LVEF: Left ventricular ejection fraction
CO: Cardiac output
E: Early diastolic velocity of mitral inflow
A: Late diastolic velocity of mitral inflow
e′: Early diastolic mitral annular velocity
CVVH: Continuous veno-venous hemofiltration
CVVHDF: Continuous veno-venous hemodiafiltration
PSW: Propensity score weighting
sIPTW: Stabilized inverse probability of treatment weighting
CRS: Cardiorenal syndrome
HF: Heart failure
IL: Interleukin
TNF: Tumor necrosis factor
MV: Mechanical ventilation
