# Supplementary material for: Clinical outcomes of severe sepsis and septic shock patients with left ventricular dysfunction undergoing continuous renal replacement therapy
Source: Sci Rep. 2022 Jun 7;12:9360. doi: 10.1038/s41598-022-13243-9 (PMC9174253; doi:10.1038/s41598-022-13243-9)
Supplement: Supplementary file 1 — Supplementary Table S1. [file 41598_2022_13243_MOESM1_ESM.docx]

Table S1. Patient characteristics between three centers [at baseline and at the start of continuous renal replacement therapy (CRRT)]

| Characteristics | Center 1  (n = 39) | Center 2  (n = 46) | Center 3  (n = 47) | *P* |
| --- | --- | --- | --- | --- |
| Age, years | 61.54 ± 9.93 | 62.24 ± 10.47 | 64.13 ± 8.46 | 0.429 |
| Male, n (%) | 24 (61.54) | 35 (76.09) | 31 (65.96) | 0.329 |
| BMI, kg/m^2^ | 24.79 ± 3.28 | 24.2 ± 3.35 | 24.76 ± 3.25 | 0.633 |
| MAP, mmHg | 82.03 ± 5.18 | 82.72 ± 5.31 | 82.91 ± 5.69 | 0.735 |
| Laboratory tests |  |  |  |  |
| Leukocyte count, ×10^9^/L | 13.91 ± 3.68 | 15.37 ± 3.14 | 15.16 ± 3.75 | 0.131 |
| Neutrophil percentage, % | 80.66 ± 7.47 | 82.71 ± 6.80 | 79.67 ± 7.82 | 0.134 |
| PLT count, ×10^9^/L | 178.05 ± 57.73 | 181.33 ± 57.29 | 175.4 ± 52.52 | 0.877 |
| Blood pH | 7.33 ± 0.06 | 7.34 ± 0.05 | 7.34 ± 0.07 | 0.668 |
| Baseline creatinine, mg/dL | 0.9 ± 0.29 | 1.02 ± 0.32 ^c^ | 0.87 ± 0.25 ^c^ | 0.031 |
| Serum potassium, mEq/L | 4.72 ± 0.69 | 4.83 ± 0.61 | 4.85 ± 0.72 | 0.609 |
| Baseline eGFR, mL/min/1.73m^2^ | 82.52 ± 23.3 | 75.51 ± 21.95 | 83.47 ± 21.42 | 0.178 |
| BUN, mg/dL | 15.89 ± 3.12 | 16.42 ± 2.42 | 15.5 ± 3.4 | 0.309 |
| CK-MB, IU/L | 24.26 ± 20.83 | 26.67 ± 22.19 | 20.96 ± 12.7 | 0.345 |
| ALT, U/L | 53.33 ± 44.64 | 73.91 ± 76.05 ^c^ | 36.32 ± 21.23 ^c^ | 0.002 |
| Total bilirubin, mg/dL | 1.29 ± 0.69 | 1.5 ± 1.26 ^c^ | 0.95 ± 0.63 ^c^ | 0.010 |
| Lactate, mg/dL | 72.26 ± 48.81 | 65.36 ± 38.98 | 57.91 ± 34.78 | 0.269 |
| Hb, g/L | 132.44 ± 12.93 | 132.48 ± 12.09 | 130.68 ± 12.52 | 0.736 |
| Six-hour UO at admission, mL | 446.38 ± 154.67 | 424.5 ± 147.28 | 459.15 ± 136.54 | 0.514 |
| Primary diagnosis |  |  |  |  |
| Pneumo-sepsis, n (%) | 16 (41.03) | 9 (19.57) | 17 (36.17) | 0.077 |
| Urosepsis, n (%) | 3 (7.69) | 6 (13.04) | 3 (6.38) | 0.502 |
| Abdominal sepsis, n (%) | 12 (30.77) | 17 (36.96) | 17 (36.17) | 0.814 |
| Other cause, n (%) | 8 (20.51) | 14 (30.43) | 10 (21.28) | 0.477 |
| Type of surgery |  |  |  |  |
| Abdominal surgery, n (%) | 7(17.95) | 2(4.35) | 3(6.38) | 0.116 |
| Urinary surgery, n (%) | 1(2.56) | 1(2.17) | 0(0) | 0.534 |
| Others, n (%) | 3(7.69) | 1(2.17) | 1(2.13) | 0.439 |
| Postoperative, n (%) | 11 (28.21) ^a,b^ | 4 (8.7) ^a^ | 4 (8.51) ^b^ | 0.014 |
| Positive blood culture, n (%) | 15 (38.46) | 21 (45.65) | 19 (40.43) | 0.781 |
| Invasive MV, n (%) | 24 (61.54) | 26 (56.52) | 29 (61.7) | 0.850 |
| Comorbidities |  |  |  |  |
| Hypertension, n (%) | 10 (25.64) | 11 (23.91) | 12 (25.53) | 0.978 |
| Diabetes mellitus, n (%) | 7 (17.95) | 6 (13.04) | 8 (17.02) | 0.800 |
| Coronary artery disease, n (%) | 2 (5.13) | 5 (10.87) | 1 (2.13) | 0.190 |
| Heart failure, n (%) | 6 (15.38) | 3 (6.52) | 7 (14.89) | 0.351 |
| Medication at admission |  |  |  |  |
| Noradrenaline, n (%) | 31 (79.49) | 36 (78.26) | 30 (63.83) | 0.173 |
| Dopamine, n (%) | 13 (33.33) | 10 (21.74) | 6 (12.77) | 0.072 |
| Glucocorticoid, n (%) | 3 (7.69) | 9 (19.57) | 7 (14.89) | 0.297 |
| Digitalis during hospitalization, n (%) | 8(20.51) | 7(15.22) | 6(12.77) | 0.612 |
| Resuscitation fluid in first 36 hours (crystal solution), mL | 3144.87 ± 481.34 | 3047.83 ± 445.71 | 3038.30 ± 420.95 | 0.491 |
| Echocardiography |  |  |  |  |
| LVEDD, mm | 49.92 ± 2.3 | 48.86 ± 3.15 | 48.65 ± 2.81 | 0.088 |
| LVESD, mm | 36.71 ± 3.18 ^b^ | 36.09 ± 3.72 ^c^ | 34.29 ± 3.03 ^b,c^ | 0.002 |
| CO, L/min | 4.07 ± 0.83 | 3.87 ± 0.92 | 4.07 ± 0.75 | 0.451 |
| LVEF, % | 48.59 ± 12.26 | 49.49 ± 13.61 | 54.14 ± 12.17 | 0.090 |
| E, m/s | 0.75 ± 0.25 | 0.77 ± 0.24 | 0.79 ± 0.21 | 0.664 |
| A, m/s | 0.7 ± 0.16 ^b^ | 0.79 ± 0.19 | 0.8 ± 0.18 ^b^ | 0.025 |
| E/A | 1.07 ± 0.29 | 1 ± 0.3 | 1.02 ± 0.32 | 0.632 |
| e′, m/s | 0.08 ± 0.02 ^b^ | 0.08 ± 0.03 ^c^ | 0.05 ± 0.01 ^b,c^ | < 0.001 |
| E/e' | 9.46 ± 3.77 ^b^ | 9.85 ± 3.02 ^c^ | 15.70 ± 6.02 ^b,c^ | < 0.001 |
| Cardiac function |  |  |  | < 0.001 |
| Systolic dysfunction, n (%) | 19 (48.72) ^b^ | 18 (39.13) ^c^ | 0 (0) ^b,c^ |  |
| Diastolic dysfunction, n (%) | 17 (43.59) ^b^ | 21 (45.65) ^c^ | 33 (70.21) ^b,c^ |  |
| Systolic and diastolic dysfunction, n (%) | 3 (7.69) ^b^ | 7 (15.22) | 14 (29.79) ^b^ |  |
| CRRT protocol |  |  |  |  |
| Mode |  |  |  | 0.626 |
| CVVH | 16 (41.03) | 15 (32.61) | 15 (31.91) |  |
| CVVHDF | 23 (58.97) | 31 (67.39) | 32 (68.09) |  |
| Anticoagulant |  |  |  | 0.772 |
| Sodium citrate | 20 (51.28) | 20 (43.48) | 22 (46.81) |  |
| Heparin | 19 (48.72) | 26 (56.52) | 25 (53.19) |  |
| Blood flow, mL/min | 149.36 ± 17.37 | 155.43 ± 18.91 | 152.13 ± 17.34 | 0.297 |
| CRRT dose, mL/kg/h | 26.95 ± 2.35 ^b^ | 26.89 ± 2.74 ^c^ | 30.38 ± 3.27 ^b,c^ | <0.001 |
| Ultrafiltration dose, mL/kg/h | 27.05±2.09 | 27.33±2.16 | 26.89±1.76 | 0.576 |
| Dialysis dose, mL/kg/h | 15.09±1.76 ^a,b^ | 18.10±3.59 ^a^ | 20.03±3.44 ^b^ | <0.001 |
| SOFA scores | 12.36 ± 1.93 | 12.37 ± 1.79 | 12.51 ± 2 | 0.915 |
| APACHE II scores | 24.46 ± 3.02 | 24.93 ± 3.19 | 24.79 ± 3.44 | 0.793 |
| At the start of CRRT |  |  |  |  |
| Noradrenaline, n (%) | 39 (100) ^a,b^ | 38 (82.61) ^a,c^ | 25 (53.19) ^a,c^ | < 0.001 |
| Noradrenaline, µg/kg/min | 0.32 ± 0.19 | 0.31 ± 0.22 | 0.22 ± 0.21 | 0.132 |
| Six-hour UO before CRRT initiation, mL | 413.79 ± 157.93 ^a,b^ | 239.2 ± 116.97 ^a,c^ | 144.04 ± 61.24 ^b,c^ | < 0.001 |
| Total duration CRRT, hour | 82.56 ± 26.4 | 84.28 ± 19.14 | 87.64 ± 19.89 | 0.554 |
| Creatinine, mg/dL | 1.05 ± 0.31 ^a,b^ | 1.78 ± 1.04 ^a,c^ | 2.71 ± 0.95 ^b,c^ | < 0.001 |
| MAP, mmHg | 80.54 ± 5.3 ^b^ | 81.7 ± 8.1 ^c^ | 92.4 ± 11.22 ^b,c^ | < 0.001 |
| SOFA | 12.69 ± 1.72 | 13.07 ± 1.64 | 13.3 ± 1.83 | 0.273 |
| APACHE II | 26.56 ± 3.8 ^a,b^ | 29.15 ± 4.43 ^a^ | 30.04 ± 4.62 ^b^ | 0.001 |
| At the end of CRRT |  |  |  |  |
| Noradrenaline, n (%) | 19 (48.72) | 22 (47.83) | 19 (40.43) | 0.687 |
| Noradrenaline, μg/kg/min | 0.33 ± 0.14 ^b^ | 0.26 ± 0.11 ^c^ | 0.16 ± 0.09 ^b,c^ | <0.001 |
| Creatinine, mg/dL | 1.00 ± 0.57 ^a,b^ | 1.53 ± 1.01 ^a^ | 1.70 ± 0.89 ^b^ | <0.001 |
| MAP, mmHg | 95.95 ± 16.52 | 94.28 ± 12.54 | 96.72 ± 15.68 | 0.725 |
| SOFA | 10.31 ± 4.95 | 7.39 ± 6.19 | 9.57 ± 5.71 | 0.054 |
| APACHE II | 20.82 ± 8.57 | 16.91 ± 9.96 | 17.83 ± 8.68 | 0.124 |

Note:

Abbreviations: BMI, body mass index; MAP, mean arterial pressure; PLT, platelet; eGFR, estimated glomerular filtration rate; BUN, blood urea nitrogen; CK-MB, creatine kinase myocardial band; Hb, hemoglobin; UO, urine output; MV, mechanical ventilation; LVEED, left ventricular end-diastolic diameter; LVESD, left ventricular end-systolic diameter; CO, cardiac output; LVEF, left ventricular ejection fraction; E, peak velocity of early diastolic trans-mitral flow; A, peak velocity of late trans-mitral flow; e’, peak velocity of early diastolic mitral annular motion; CVVH, continuous veno-venous hemofiltration; CVVHDF, continuous veno-venous hemodiafiltration; SOFA, Sequential Organ Failure Assessment; APACHE II, Acute Physiology And Chronic Health Evaluation II.

The differences of patient characteristics among the three centers were compared by One-Way ANOVA method or Welch analysis based on the homogeneity of variance test for continuous variables, or Chi-square test or Fisher exact test for categorical variables. Post-hoc test for pairwise comparisons to compare centers with each other were performed at a significance level of 5% with Bonferroni adjustment.

^a^ center 1 vs. center 2: Bonferroni adjustment P<0.05

^b^ center 1 vs. center 3: Bonferroni adjustment P<0.05

^c^ center 2 vs. center 3: Bonferroni adjustment P<0.05
