# Supplementary material for: Clinical outcomes of severe sepsis and septic shock patients with left ventricular dysfunction undergoing continuous renal replacement therapy
Source: Sci Rep. 2022 Jun 7;12:9360. doi: 10.1038/s41598-022-13243-9 (PMC9174253; doi:10.1038/s41598-022-13243-9)
Supplement: Supplementary file 3 — Supplementary Information 2. [file 41598_2022_13243_MOESM3_ESM.docx]

The positivity assumption of propensity weighting method was examined. In order to avoid that the subjects in the treatment group with lower propensity score and the control group with higher propensity score would obtain a large weight and ensure that the weight has a certain stability, we used marginal probability adjustment in the IPTW analysis, which reduced the possibility of extreme weight.

Propensity weighting methods require fulfillment of the positivity assumption that every patient must have a nonzero probability to receive either treatment. The propensity score overlap between two groups was 0.397 in the present study.
